# The mechanisms linking perceived stress to pilots’ safety attitudes: a chain mediation effect of job burnout and cognitive flexibility

**DOI:** 10.3389/fpubh.2024.1342221

**Published:** 2024-06-04

**Authors:** Zhao Yanzeng, Zhu Keyong, Cai Hongmin, Liu Ziyu, Luo Pengyu, Wang Lijing

**Affiliations:** ^1^Fundamental Science on Ergonomics and Environment Control Laboratory, School of Aeronautic Science and Engineering, Beihang University, Beijing, China; ^2^Beijing Advanced Innovation Centre for Biomedical Engineering, School of Engineering Medicine, Beihang University, Beijing, China; ^3^Zhuhai Xiangyi Aviation Technology Co., Ltd., Zhuhai, China

**Keywords:** perceived stress, safety attitudes, job burnout, cognitive flexibility, pilots, mediation effect

## Abstract

**Introduction:**

Pilots’ safety attitude is crucial for aviation safety. Current research shows a correlation between perceived stress and safety attitude, yet the mechanism underlying this association remains unclear. Against the backdrop of heightened attention to pilots’ stress, this study aims to thoroughly explore the inherent connection between pilot safety attitudes and their perceived stress, offering targeted insights into preventing and addressing safety attitude issues arising from pilot stress.

**Methods:**

Through path analysis of questionnaire data from 106 civil aviation pilots in China, this study systematically investigates the roles of job burnout and cognitive flexibility in the relationship between perceived stress and safety attitude. The study reveals the chain-mediated mechanism of these two factors.

**Results:**

The results demonstrate a significantly negative correlation between pilots’ perceived stress and safety attitude, with cognitive flexibility and job burnout fully mediating this relationship, and cognitive flexibility affecting job burnout. A detailed analysis of the three dimensions of job burnout reveals varying impacts of emotional exhaustion, depersonalization, and reduced personal accomplishment on the aforementioned path. The research model exhibits a good fit (GFI=0.902), providing new theoretical perspectives on the association between pilots’ perceived stress and safety attitude.

**Discussion:**

The findings offer practical implications for improving pilots’ safety attitude by proposing targeted measures to alleviate the adverse impacts of perceived stress on safety attitude, thereby promoting aviation safety.

## Highlights

- Innovative Perspectives & Holistic Exploration: This study unveils that the relationship between pilots’ perceived stress and safety attitudes is not a straightforward causal link but rather intricately influenced by other variables. Approaching the issue through the lenses of job burnout and cognitive flexibility, our research systematically investigates the roles of these factors in the nexus between perceived stress and safety attitudes. This provides a novel theoretical perspective, expanding our comprehension of the mechanisms shaping pilots’ psychological well-being and safety attitudes.
- Empirical Validation of Amplification Effects: By delineating the three dimensions of job burnout, our research empirically substantiates the transmission and amplification effects of stress. Quantifying the inherent logic in the connections among perceived stress, cognitive flexibility, job burnout, and safety attitudes contributes to advancing the corresponding theories.
- Practical Insights for Augmented Safety: Proposing the mediating roles of job burnout and cognitive flexibility offers practical insights for enhancing pilot safety attitudes. Recommendations for airlines include strengthening organizational support, improving working conditions, providing positive feedback, and conducting cognitive training programs to alleviate the impact of perceived stress on safety attitudes.

## 1 Introduction

In the aviation industry, flight safety stands as a paramount concern. One central to this is the safety attitudes of pilots, which encompass their beliefs about safety’s importance and their motivation to act upon these beliefs (1). Previous studies have underscored the pivotal role of these attitudes in shaping pilots’ risk perception, prioritizing safety during emergencies, and influencing decision-making (2). Negative safety attitudes were pinpointed as key contributors to aviation accidents and precursor events (3). Conversely, positive safety attitudes bolster pilots’ professional pride and standardize safety procedures, leading to a decrease in unsafe behaviors and the likelihood of accidents (4, 5).

Given that attitudes significantly shape behavior, understanding these underlying beliefs is essential when aiming to drive behavioral change (6). However, current research has yet to delve into the factors shaping pilots’ safety attitudes, leaving a critical knowledge gap that hampers effective strategies for enhancing flight safety.

To bridge this gap, this study seeks to elucidate the pathways influencing pilot safety attitudes, offering both theoretical and empirical foundations for designing more impactful safety training and educational programs. By conducting a comprehensive literature review, empirical investigations, and quantitative data analysis, this study aims to uncover the mechanisms that shape these attitudes. These findings aspire to introduce fresh perspectives and methodologies to aviation safety management, with a specific focus on reducing accidents through the enhancement of pilots’ safety attitudes. This study holds significant implications for airlines, safety management professionals, and policymakers striving to elevate safety standards within the aviation industry.

### 1.1 Relationship between perceived stress and safety attitude

Stress encompasses the various demands and expectations individuals encounter in their work, accompanied by corresponding psychological and physiological responses (7). As a subjective experience, perceived stress can vary widely among individuals exposed to the same work environment and job requirements. Within the pilot profession, stress emerges as a pervasive factor influenced by diverse elements such as family issues, social stressors, career uncertainties, aircraft accidents, and demanding flight schedules. While moderate levels of stress can help maintain pilots’ focus, excessive stress can impair performance. When the underlying causes of perceived stress remain unaddressed, stress tends to accumulate, leading to persistent and escalating levels. This chronic stress can detrimentally affect commercial pilots both physiologically and psychologically, significantly compromising their flying capabilities (8).

Research has shown that pilots who fail to cope with stress may become depressed or even self-destructive. They may externalize their feelings and show them, or blame their misfortunes on others. When pilots encounter unexpected situations, those who perceive high levels of stress may exhibit “warning signals,” such as defensiveness, arrogance, hostility, deterioration in pilot performance, or increased risk-taking (9). Other studies have also revealed a negative correlation between pilots’ perceived stress and their safety attitudes through quantitative data (10–12).

However, despite existing research on the correlation between a pilot’s safety attitude and their perceived stress, the relationship between perceived stress and safety attitude is not a straightforward cause-and-effect scenario. In this process, the influence of buffering variables remains unexplored, and the specific psychological mechanisms through which pilot stress affects their safety attitude have yet to be elucidated.

Research has demonstrated that higher stress levels are perceived by pilots in comparison to individuals engaged in alternative occupations (13). To effectively mitigate the risks of aviation accidents stemming from safety attitudes, it is crucial to delve into the path relationship between a pilot’s perceived stress and their safety attitude, examining the impact pathways and mechanisms through which stress influences safety attitudes and identifying key mediating variables. This comprehensive exploration is not only beneficial for unveiling the psychological processes through which stress affects safety attitudes but also provides a basis for aviation companies and training institutions.

Building upon this, the first hypothesis proposed in this study is as follows:

*H1*: There is a significant negative correlation between a pilot's perceived stress and their safety attitude.

### 1.2 Mediating role of job burnout

The concept of job burnout was initially introduced by Freudenberger in psychology to describe a state of overwhelming exhaustion when work excessively demands an individual’s capabilities, energy, and resources (14). It was subsequently divided into three dimensions by Maslach and Jackson: emotional exhaustion, depersonalization, and reduced personal accomplishment (15).

Compared to individuals with low levels of job burnout, those with high levels are more susceptible to the spiral of resource depletion and are observed to conserve their limited energy to avoid further resource loss (16, 17). According to the Conservation of Resources theory, individuals are believed to possess finite personal resources and, driven by an innate self-protective instinct, are driven to acquire, preserve, and maintain these resources. A continuous drain on their emotional and mental resources is acutely felt by individuals experiencing high levels of job burnout, particularly when they perceive a deficit in personal resources, which may lead them to refrain from investing additional resources in maintaining safety, thereby leading to non-compliance with safety regulations, neglect of safety procedures, and disregard for safety risks.

Furthermore, the Job Demand-Resource theory (18), along with quantitative studies on employees (19)and nurses (20), has indicated that stressors such as work overload, lack of autonomy, emotional demands, low social support, and role ambiguity can result in feelings of exhaustion and the development of negative, callous attitudes toward work (21). When the work demands perceived surpass the available resources, heightened levels of perceived stress are experienced by individuals, who not only confront the challenges of their job but also grapple with additional psychological burdens. As a result, these individuals are more prone to fatigue and resource depletion, culminating in job burnout (22, 23).

For pilots, increased perceived stress and work demands can lead to the onset of job burnout. Recognizing signs of burnout, pilots may become more sensitive to resource depletion. Thus, when pilots are engaged in activities that consume more of their precious physical and cognitive resources, such as implementing spontaneous safety changes and safety suggestions while striving to complete flight tasks (24), they are more inclined to adopt a passive safety attitude of neglecting or avoiding safety behaviors, including reducing work enthusiasm and lowering work engagement, among other things.

Hence, this study proposes the hypotheses:

*H2a*: Pilots’ job burnout is positively correlated with their safety attitudes.

*H2b*: Pilots’ job burnout plays a mediating role in the relationship between their perceived stress and safety attitudes.

### 1.3 Mediating role of cognitive flexibility

Cognitive flexibility, a core component of executive function, plays a pivotal role in enabling individuals to swiftly adjust and adapt cognitive strategies when confronted with new information or environments (25). This capability allows individuals to transition between different cognitive states, facilitating adept navigation of complex and ever-changing situations (26). Given that pilots require not only proficient flying skills but also the ability to make informed decisions based on specific contexts and maintain situational awareness, cognitive flexibility emerges as a highly prized attribute among pilots (27, 28).

Research has indicated that individuals with high cognitive flexibility exhibit proactive thinking, suggesting multiple solutions when facing intricate challenges rather than avoiding them (29). This proactive approach correlates with enhanced adaptability and superior coping strategies (30). Moreover, individuals with high cognitive flexibility typically demonstrate stronger social and cognitive abilities, fostering more effective communication and collaboration with peers. In contrast, those with low cognitive flexibility often display diminished social interaction skills and cognitive proficiency (31). For instance, a study involving emergency service personnel revealed that individuals with high cognitive flexibility remain mentally agile and alert, capturing diverse cues in their environment. This heightened awareness correlates with elevated safety attitudes and situational awareness, effectively mitigating unsafe behaviors and conspicuous operational errors (32).

Furthermore, cognitive flexibility has been observed to be influenced by perceived stress (33). High-stress environments pose challenges for individuals in task-switching and adapting to new circumstances, primarily due to stress-induced impairment of key prefrontal cortex regions responsible for executive functions like working memory and attention (34, 35). Studies on nursing populations have also corroborated a negative correlation between stress levels and cognitive flexibility (36), suggesting that higher stress levels are associated with decreased cognitive flexibility.

Given the aforementioned findings, it can be surmised that perceived stress may impact pilots’ safety attitudes through the mediating effect of cognitive flexibility. Elevated perceived stress levels could impair cognitive flexibility, hindering pilots’ ability to swiftly adapt cognitive strategies when faced with complex and novel flight situations. This compromised cognitive flexibility may diminish pilots’ alertness and coping capabilities, potentially resulting in decision-making errors or unsafe behaviors during operations.

Based on this, the study proposes the following hypotheses:

*H3a*: Pilot cognitive flexibility is positively correlated with their safety attitudes.

*H3b*: Pilot cognitive flexibility plays a mediating role in the relationship between their perceived stress and safety attitudes.

### 1.4 Chain-mediation role of flexibility and job burnout

Cognitive flexibility refers to an individual’s ability to process information, adapt to changes, and solve problems. It entails the capability to quickly and effectively adjust cognitive strategies across various tasks and environments. Job burnout, on the other hand, is characterized by physical and mental exhaustion stemming from prolonged work pressure and psychological strain, potentially resulting in diminished interest and enthusiasm toward work.

According to behavioral regulation theory, individuals engage in goal-oriented behavior through a series of cognitive processes, encompassing goal setting, selection, internal and external orientation, planning, execution monitoring, and feedback processing. A decline in cognitive flexibility can pose several challenges for individuals. Firstly, it may hinder their ability to set clear work goals or select appropriate ones, thereby affecting planning and execution monitoring. Secondly, individuals may struggle to process work feedback and find it challenging to adapt their behavior accordingly. Consequently, reduced cognitive flexibility can render work objectives ambiguous, leading to diminished motivation and direction in work tasks. This situation can contribute to the onset of job burnout, making it difficult for individuals to cope effectively with work challenges (37). Empirical research corroborated this perspective, revealing a correlation between job burnout and impaired executive function (38). This suggests that prolonged occupational stress may compromise cognitive function, subsequently influencing individual job burnout and safety attitudes.

When pilots confront high levels of work pressure and challenges, their cognitive abilities may suffer, resulting in reduced efficiency in executing control and difficulties in organizing and regulating their actions effectively. As a result, diminished cognitive flexibility can make pilots more vulnerable to feeling overwhelmed and experiencing job burnout. This can lead them to overlook safety considerations and develop negative safety attitudes.

Therefore, this study proposes the following hypothesis:

*H4*: Cognitive flexibility and job burnout play a chain-mediated role in the relationship between perceived stress and safety attitudes.

## 2 Methods

### 2.1 Participants

This research focuses on conducting a survey study among Chinese civil aviation pilots, employing a random sampling approach. Researchers initially developed the research questionnaire as an online electronic survey and generated a QR code for distribution. The questionnaire included variable questions mentioned in the literature review section as well as demographic questions. Between September 21, 2023, and November 1, 2023, researchers randomly selected a class of pilots undergoing recurrent training and presented the QR code to invite pilots to participate anonymously in the survey. A total of 137 pilot responses were collected for this study, of which 106 pilot data were analyzed after the removal of outliers. It is noteworthy that all participants in this survey were male, consistent with the disproportionately low representation of female pilots in the Chinese civil aviation industry (39). Ethical approval for this research was obtained from the Ethics Committee of the author’s institution, and all procedures were conducted by relevant guidelines and regulations.

Table 1 presents the basic demographic information of the participants. The data collection showed a nearly 2:1 ratio between First officers and captains/instructors, aligning with the typical distribution of pilots in China. This suggests that the collected data to some extent represents the Chinese pilot population. Additionally, the data predominantly falls within the 21–40 age range, with an average age of 33.593 ± 7.702 years. The distribution of flight experience aligns with the distribution of professional titles among pilots.

**Table 1:** Demographics of participants (*n* = 106).

	Group	*N*	%
Post	First Officer	59	55.7
	Captain	25	23.6
	Instructor	22	20.8
Age	21–30	49	46.2
	31–40	41	38.7
	40+	16	15.1
Flight experience	0–3,000	44	41.5
	3,001–7,000	26	24.5
	7,000+	36	34.0
Total		106	100

### 2.2 Self-reported scales

#### 2.2.1 Pilot safety attitude questionnaire

Pilot safety attitudes are reflected in their performance in training, and work, as well as in communication and collaboration with other pilots. Based on a literature review (40) and in-depth interviews with flight management personnel from a certain airline, this study modified the safety attitude evaluation indicator system of the airline. After removing inappropriate questions, five items were retained to measure pilots’ safety attitudes. These items are as follows: (1) In daily operations, I voluntarily report safety hazards; (2) In daily operations, I actively provide safety suggestions to the company; (3) In daily operations, I frequently communicate safety information with others; (4) In daily operations, I actively learn flight-related knowledge from various sources; (5) In training, I proactively identify and address weaknesses in my skills for training and improvement. The 5-point Likert scoring method was used in the questionnaire. The higher the score, the better the safety attitude. The Cronbach’s alpha for this measurement was 0.809, indicating a high level of internal consistency. For the validity analysis of the questionnaire, based on the single-factor structure analysis, the factor loadings for all items were greater than 0.4. The KMO value was 0.745, and the *p*-value in Bartlett’s test of sphericity was 0.000.

#### 2.2.2 Perceived stress questionnaire

In a stress study specific to civil aviation pilots, which integrates the OSI (Occupational Stress Inventory) occupational stress measurement indicator system, the sources of pilots’ perceived stress were categorized as the job itself, career development, interpersonal relationships, work–family conflict, and organizational factors (7). The questionnaire utilized a five-point scale for self-assessment, where scores from 1 to 5 represent the degree of conformity from “completely inconsistent” to “completely consistent.” Higher scores indicate greater perceived stress, while lower scores suggest lower perceived stress. For the reliability and validity analysis of the questionnaire, all questions were combined into one dimension. The overall reliability of Cronbach’s alpha was 0.956. Additionally, the factor loadings for all items on each dimension were greater than 0.4. The KMO value was 0.852, and the *p*-value in Bartlett’s test of sphericity was 0.000.

#### 2.2.3 Job burnout questionnaire

Maslach’s three-dimensional model defines burnout as a psychological syndrome resulting from the job itself, encompassing the dimensions of emotional exhaustion, depersonalization, and reduced personal accomplishment. The MBI-GS scale is provided as a quantitative measurement tool. This study adheres to the 7-point self-assessment format employed by the scale, with scores ranging from 1 to 7. A score of 1 represents “never,” 2 corresponds to “a few times or less per year,” 3 to “once a month or less,” 4 to “a few times a month,” 5 to “once a week,” 6 to “a few times a week,” and 7 to “every day.” Higher scores indicate stronger burnout. The Cronbach’s alpha for each sub-dimension in this questionnaire was 0.948, 0.836, and 0.803, with a total Cronbach’s alpha of 0.903. For the validity analysis of the questionnaire, the KMO values for each dimension were 0.900, 0.740, and 0.796, and the *p*-values in Bartlett’s test of sphericity were all 0.000.

#### 2.2.4 Cognitive flexibility questionnaire

Cognitive flexibility refers to an individual’s ability to freely change their cognitive approach in different situations, adapting to environmental changes and demands. Martin & Rubin developed the Cognitive Flexibility Inventory (CFI) to assess the flexibility of an individual’s cognitive adaptation. The scale employs a 5-point self-assessment format, with 1 indicating “strongly disagree” and 5 indicating “strongly agree.” Higher scores reflect greater cognitive flexibility. The Cronbach’s alpha for the measurement in this questionnaire was 0.794. For the validity analysis of the questionnaire, based on the single-factor structure analysis, the factor loadings for all items were greater than 0.4. The KMO value was 0.851, and the *p*-value in Bartlett’s test of sphericity was 0.000.

### 2.3 Model hypothesis

**Figure 1:**
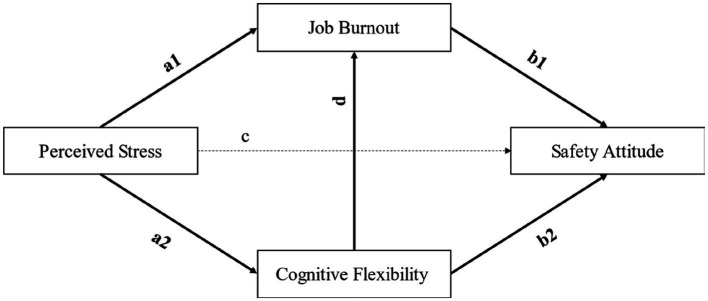
Path analysis of the hypothesis model.

As shown in Figure 1, based on the hypotheses from the literature review, the mathematical expressions of the main models in this study were summarized as follows.

$$\begin{array}{c} \mathrm{Safety attitude}=b1^{*}\;\mathrm{Job}\;\mathrm{burnout}+b2^{*}\;\mathrm{Cognitive flexibility}\\ +c^{*}\;\mathrm{Perceived stress}+e1 \end{array}$$

$$\mathrm{Job}\;\mathrm{burnout}=a1^{*}\;\mathrm{Perceived}\;\mathrm{stress}+d^{*}\;\mathrm{Cognitive}\;\mathrm{flexibility}+e2$$

$$\mathrm{Cognitive}\;\mathrm{flexibility}=a2^{*}\;\mathrm{Perceived}\;\mathrm{stress}+e3$$

### 2.4 Data processing

To mitigate the potential issue of common method bias, the present study initially employed Harman’s single-factor test for the detection of common method bias. Subsequently, this research utilized correlation analysis to discern the relationships among various factors as mentioned in the literature review section, while also examining the presence of multicollinearity issues. Following this, stepwise regression analysis was employed to ascertain the existence and direction of the effects of different variables on pilots’ safety attitudes. Finally, path analysis was conducted to explore the intricate relationships among variables, aiming to gain a deeper understanding of the mechanism underlying the formation of safety attitudes. All data analyses were processed through SPSS AU.

## 3 Results

### 3.1 Common method bias test

The results indicated that the first unrotated factor accounted for 29.720% of the total variance, which was less than 40% of the total variance explained (41). Accordingly, this study did not demonstrate a significant common method bias.

### 3.2 Descriptive statistics and correlation analysis of variables

The results of the correlation analysis are presented in Table 2. A significant negative correlation was observed between safety attitude and perceived stress (−0.330**). Similarly, job burnout was found to correlate positively with perceived stress (0.672**), while it also displayed a significant negative correlation with safety attitude (−0.535**). Cognitive flexibility was significantly negatively correlated with perceived stress (−0.553**) and positively correlated with safety attitude (0.503**). Additionally, a significant negative correlation was noted between cognitive flexibility and job burnout (−0.542**). All correlations specified in the hypotheses were confirmed.

**Table 2:** Descriptive statistics and correlation coefficients.

Variables	Mean	S. D.	1	2	3	3.1	3.2	3.3	4
Safety Attitude (1)	3.732	0.606	1						
Perceived Stress (2)	2.754	0.681	−0.330**	1					
Job Burnout (3)	3.068	0.965	−0.535**	0.672**	1				
JB-Emotional Exhaustion (3.1)	3.457	1.401	−0.432**	0.520**	0.849**	1			
JB-Depersonalization (3.2)	3.049	1.244	−0.461**	0.587**	0.873**	0.699**	1		
JB-Reduced Personal Accomplishment (3.3)	2.759	0.995	−0.396**	0.516**	0.680**	0.293**	0.396**	1	
Cognitive Flexibility (4)	3.588	0.413	0.503**	−0.553**	−0.542**	−0.363**	−0.448**	−0.508**	1

*p < 0.05, **p < 0.01. The darker the color, the stronger the correlation. The lighter the color, the weaker the correlation.

### 3.3 Stepwise regression model for safety attitude

Using safety attitude as the dependent variable and perceived stress, job burnout, and cognitive flexibility as independent variables, regression analysis was conducted. The model was found to be statistically significant (*F* (2,103) = 27.824, *p* < 0.001). The statistical results revealed that perceived stress did not have a significant impact on safety attitude. However, job burnout (beta = −0.372, *p* < 0.001) and cognitive flexibility (beta = 0.302, *p* = 0.002) were observed to significantly influence safety attitude. These findings are consistent with the study’s hypotheses, suggesting that perceived stress influences safety attitude through the mediating effects of job burnout and cognitive flexibility.

### 3.4 Path analysis results

Linear regression reflects the direct relationship between independent and dependent variables. However, the relationships between variables are often intricate. It is challenging to express all these relationships with a single regression model. Path analysis is then considered.

Drawing on the literature review and the proposed hypotheses, the path model constructed in this study encompasses several routes: (1) perceived stress directly influences safety attitudes, (2) perceived stress affects safety attitudes indirectly through job burnout, (3) perceived stress impacts safety attitudes indirectly via cognitive flexibility, and (4) perceived stress affects job burnout through the mediating effect of cognitive flexibility, which in turn influences safety attitudes.

**Figure 2:**
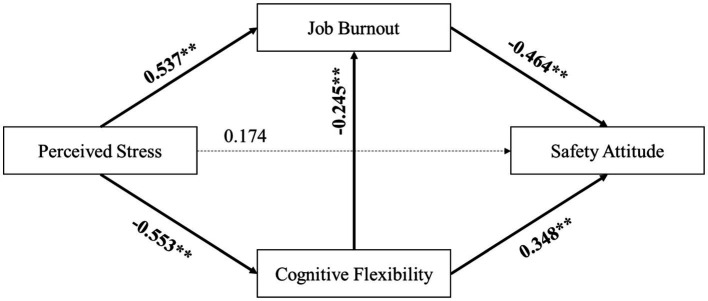
Path coefficient of the hypothesis model.

The results of the path analysis, as depicted in Figure 2, aligned with those from the stepwise regression analysis. The path from perceived stress to safety attitude was not significant, whereas all other paths were significant. These findings are in perfect agreement with the hypotheses, suggesting that perceived stress influences safety attitude through the mediating effects of cognitive flexibility and job burnout.

**Figure 3:**
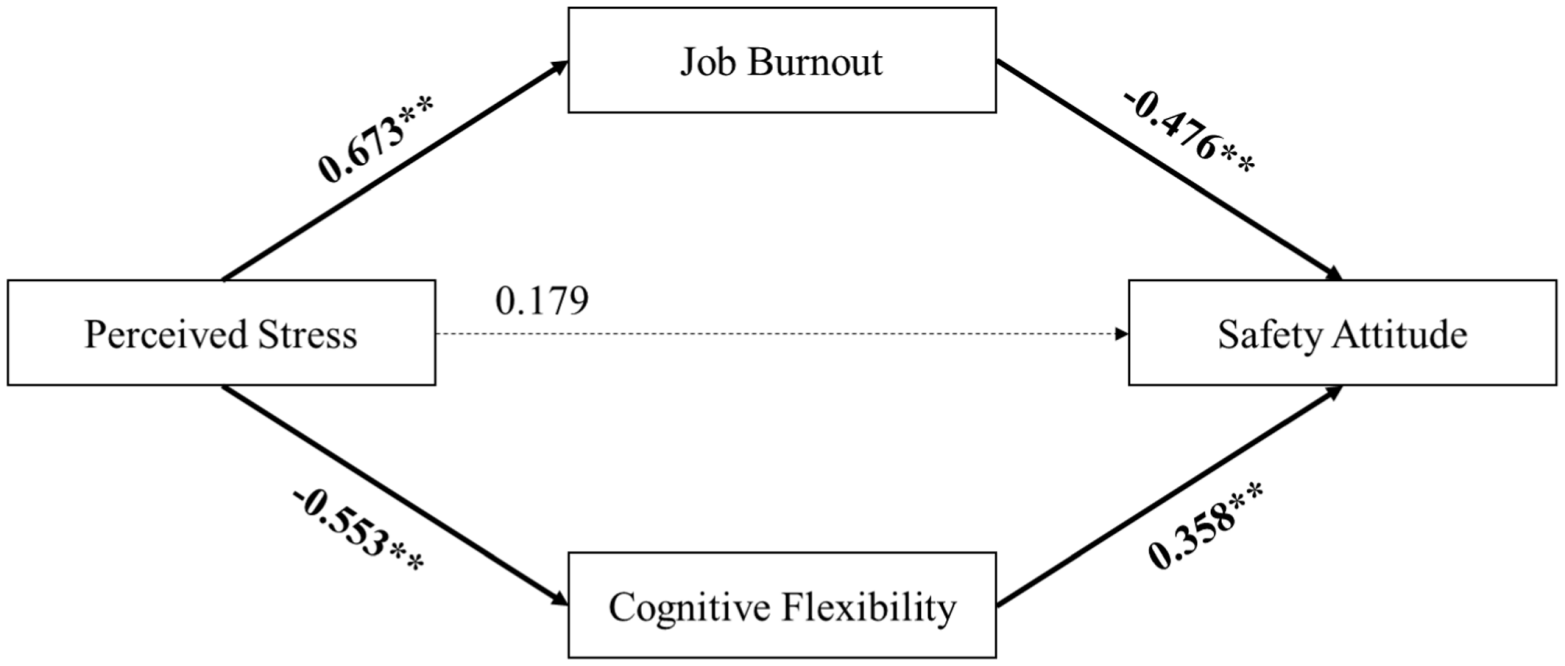
Path coefficient of the alternative model.

To further validate the reliability of this hypothesized model, the path from cognitive flexibility to job burnout was removed experimentally. As shown in Figure 3, all path coefficients remained significant, and their magnitudes were not notably different, affirming the robustness of the model. Additionally, the removal of this path led to a decrease in the model’s *R*^2^, reinforcing the notion that the chain-mediation model more accurately represents the mechanism through which perceived stress affects safety attitude.

To delve deeper into the roles played by the three dimensions of job burnout and cognitive flexibility in mediating the relationship between perceived stress and safety attitude, a comprehensive path analysis was performed on the individual dimensions of job burnout. The model’s Goodness-of-Fit Index (GFI) was 0.902, indicating a good fit for the model.

**Figure 4:**
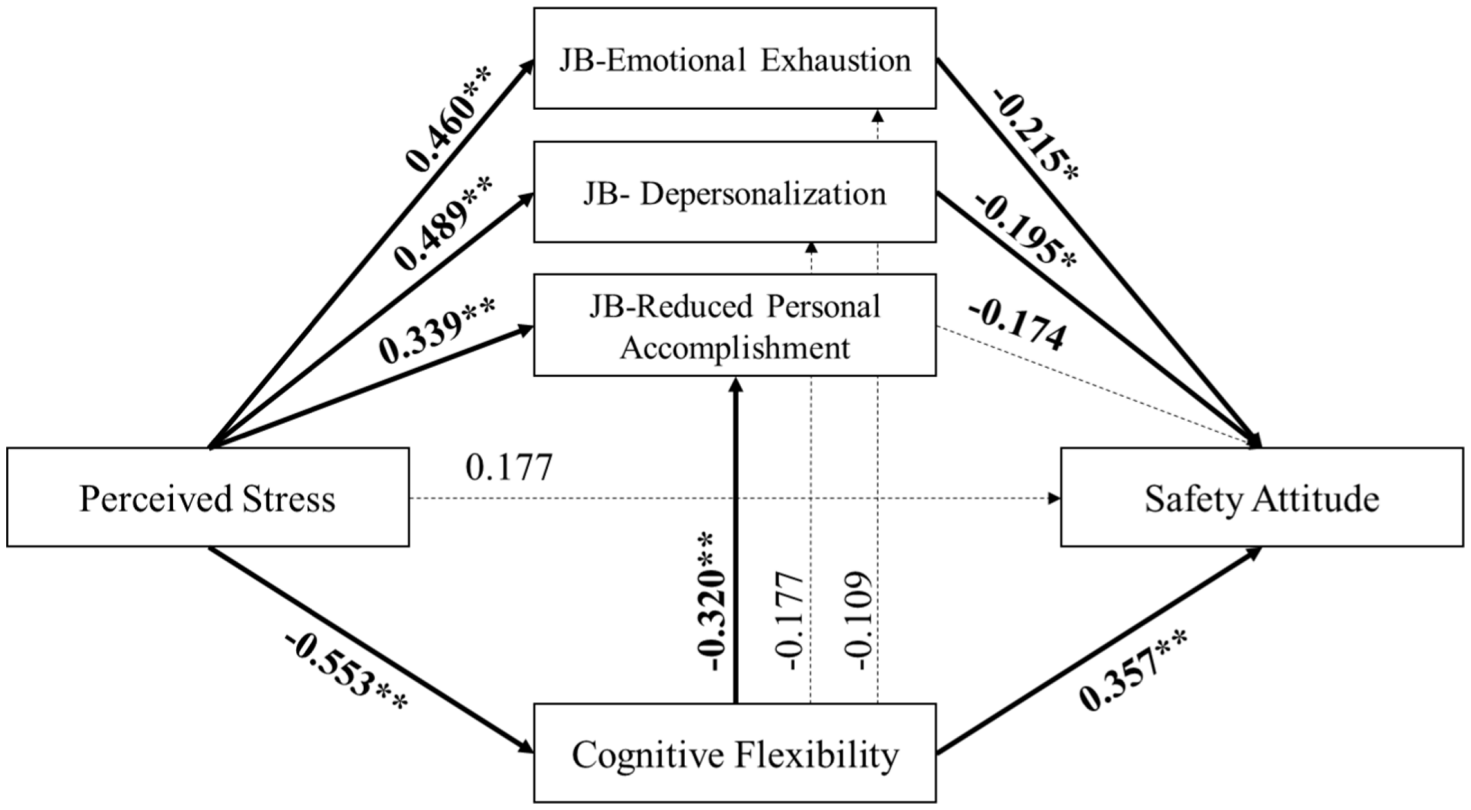
Path coefficient of the detailed model.

As illustrated in Figure 4, significant associations were found between perceived stress and the three dimensions of job burnout. Notably, emotional exhaustion and depersonalization were identified as the dimensions that significantly impact safety attitudes. Furthermore, the influence of cognitive flexibility on job burnout was predominantly mediated by the dimension of Reduced Personal Accomplishment.

## 4 Discussions

### 4.1 Negative relationship between perceived stress and safety attitude

A negative correlation between pilots’ perceived stress and safety attitude was identified in this study, which is consistent with findings from previous research (10, 11). In other words, as higher levels of stress are experienced by pilots, their safety attitudes tend to deteriorate. Similar to relationships between stress and attitude uncovered in other studies (42–44), the link between stress and safety attitude is influenced by multiple variables. Further analysis of the relationship between pilots’ perceived stress and safety attitude revealed that the impact is not directly correlated; the negative effects of perceived stress may need to go through intermediate processes before influencing safety attitude. This finding emphasizes the importance of in-depth research into the mechanisms between pilots’ perceived stress and safety attitudes. Gaining an understanding of these mechanisms is crucial so that more effective recommendations for pilot stress management can be provided. Doing so enables the targeted development of training programs that enhance a pilot’s ability to cope with high-pressure environments, thereby fostering the formation of positive safety attitudes, effectively reducing accident risks, and elevating aviation safety standards. Therefore, a thorough investigation into the relationship between pilot stress and safety attitudes holds significant theoretical and practical value for stress management and accident prevention.

### 4.2 Mediating effect of job burnout

Compared to other occupations, pilots work in a distinctive physical environment, subjecting them to increased physical exhaustion and the need to manage multiple tasks efficiently, along with various stressors related to passenger safety (27). Additionally, pilots face an overwhelming workload, which includes demanding flight training and unpredictable work schedules, among other challenges. As a group susceptible to work-related exhaustion, pilots are prone to depleting their resources excessively when faced with high job demands, heightening their susceptibility to job burnout (45–47). Previous research has shown that employees with high levels of burnout are more likely to be troubled by negative emotions and attitudes, and they tend to approach organizational safety recommendations with skepticism and a negative attitude (48, 49). Consistent with the research hypothesis, when pilots are in a state of job burnout, safety attitude, considered beyond the operational tasks of flying, diminishes in interest and enthusiasm. Content of safety attitude may be perceived as unnecessary outside their job scope, pessimistic expectations about behavioral outcomes are held, and a resource-conservative strategy to reduce their level of engagement in work-related safety behaviors tended to be adopted (16, 50), adopting an attitude of neglect or avoidance toward safety-related activities.

The detailed analysis of the three dimensions of job burnout in this study supports this explanation. Significant correlations were found between perceived stress and emotional exhaustion, depersonalization, and reduced personal accomplishment in terms of burnout. However, concerning the mediating impact on safety attitude, it is primarily emotional exhaustion and depersonalization that come into play, revealing that different dimensions of job burnout have varying effects on attitude formation (51, 52). In situations of job burnout, individuals tend to avoid additional tasks due to emotional exhaustion and a low sense of responsibility toward work (53), resulting in poorer safety attitudes. Combined with social exchange theory (54), when individuals perceive that the expected rewards are not provided by the organization, they may reduce work engagement and safety behaviors as a form of “punishment” for the organization. Therefore, to prevent pilot burnout, intervention should focus on emotional exhaustion and depersonalization, enhancing organizational support and fostering positive social exchange relationships.

### 4.3 Mediating effect of cognitive flexibility

Consistent with the hypothesis, pilots experiencing stress may encounter a decline in cognitive flexibility, impacting their cognitive processes related to flight tasks and safety concerns. Conversely, reducing perceived stress may result in improved cognitive flexibility, thereby enhancing safety attitudes. This aligns with the findings of a study on cognitive flexibility training by Fornette et al. (55). Following the training, pilots exhibited improved cognitive flexibility, which positively influenced emotional regulation and significantly enhanced flight performance scores. These results highlight the behavioral and attitudinal benefits associated with increased cognitive flexibility among pilots during uncertain flight situations (27). Considering the physiological mechanisms of cognitive flexibility (56, 57), it requires the involvement of the prefrontal cortex in regulation. Prolonged stress and negative emotions may lead to a decline in prefrontal cortex function. Consequently, pilots with low cognitive flexibility might adhere to their thinking patterns, strategies, and behaviors when facing flight pressure, making it challenging to cope with stress. This, in turn, leads to a deterioration of their psychological state, a reduction in confidence and responsibility for flight safety, and a decrease in proactive and positive attitudes toward flight safety. On the other hand, pilots with high cognitive flexibility can adapt their thinking patterns, strategies, and behaviors flexibly when facing flight pressure (27), effectively alleviating stress, maintaining a positive psychological state, enhancing confidence and responsibility for flight safety, and increasing proactive and positive attitudes toward flight safety.

Cognitive flexibility can be enhanced through cognitive-behavioral interventions (29), as studies on prefrontal functioning and posttraumatic stress disorder demonstrate increased flexibility after treatment (27). Therefore, it is possible to mitigate the adverse effects of pressure on job burnout by enhancing pilots’ cognitive flexibility.

### 4.4 Chain-mediation effect of flexibility and job burnout

The pathway from perceived stress through cognitive flexibility to job burnout, and finally to safety attitude, illustrates both the transmission and amplification effects of stress. This finding confirms Hypothesis H4, which posited that stress influences safety attitudes not only directly or indirectly but also through the chain mediation involving cognitive flexibility and job burnout. Specifically, perceived stress leads to a decrease in cognitive flexibility, rendering pilots more susceptible to job burnout, which in turn results in a deterioration of their safety attitudes.

Further analysis of the three dimensions of job burnout reveals that the influence of cognitive flexibility is predominantly evident in its impact on reduced personal accomplishment. Lower cognitive flexibility may hinder pilots’ ability to effectively manage their workloads, necessitating increased effort to perform tasks and consequently leading to lower levels of personal accomplishment. This observation aligns with the proposed rationale for the association between cognitive flexibility and job burnout (38).

Based on these insights, it is evident that interventions aimed at enhancing the safety attitudes of pilots should focus on both improving cognitive flexibility and managing job burnout. Such interventions are crucial to counteract the adverse effects of high stress levels on pilot safety attitudes.

### 4.5 Limitations

This study was conducted based on data from Chinese male pilots. In the future, the sample size can be expanded to include more countries to explore cross-cultural influences. Additionally, longitudinal research data and experimental data can be combined to further validate the paths mentioned in this study.

## 5 Conclusion

This study conducted a comprehensive exploration into the intricate mediating relationships among perceived stress, safety attitude, job burnout, and cognitive flexibility in commercial airline pilots. This research introduced, for the first time, insights into the roles of job burnout and cognitive flexibility, offering a profound understanding of the mechanisms underlying safety attitude formation among pilots.

The findings underscore the pivotal roles of job burnout and cognitive flexibility in mediating the relationship between perceived stress and safety attitude. It was specifically observed that perceived stress triggers emotional exhaustion and depersonalization, leading pilots to avoid tasks beyond their basic responsibilities. Furthermore, cognitive inflexibility contributes to a diminished sense of effectiveness in their work, culminating in feelings of low achievement. The combined influence of these factors significantly impacts pilots’ safety attitudes.

This study provides airlines with a scientific theoretical path for better understanding and managing pilots’ perceived stress and offers practical guidance for developing more effective pilot stress management strategies. In terms of enhancing pilots’ psychological resources, airlines should focus on preventing and alleviating job burnout by enhancing organizational support, improving working conditions, and providing positive work feedback. This approach effectively addresses issues related to emotional exhaustion and apathy toward work. Additionally, the study recommends that airlines strengthen cognitive training activities to enhance pilots’ cognitive abilities, enabling them to adapt to stress more flexibly, handle various work scenarios effectively, and thereby improve the proactivity and initiative of their safety attitudes.

## Data availability statement

The raw data supporting the conclusions of this article will be made available by the authors, without undue reservation.

## Ethics statement

The studies involving humans were approved by ethics committee of school of aeronautics. The studies were conducted in accordance with the local legislation and institutional requirements. The participants provided their written informed consent to participate in this study.

## Author contributions

ZY: Conceptualization, Data curation, Formal analysis, Investigation, Methodology, Software, Visualization, Writing – original draft, Writing – review & editing. ZK: Project administration, Supervision, Validation, Writing – review & editing. CH: Investigation, Software, Visualization, Writing – original draft. LZ: Investigation, Validation, Writing – review & editing. LP: Conceptualization, Writing – review & editing, Resources, Data curation, Investigation, Validation. WL: Conceptualization, Project administration, Resources, Supervision, Writing – review & editing.
